# Multi-Technique Analysis and Research on the Deterioration Features of Cave 29 in the Yungang Grottoes

**DOI:** 10.3390/ma19184003

**Published:** 2026-09-20

**Authors:** Yawei Zhang, Tianyu Zhang, Lijuan Dong

**Affiliations:** 1 School of Yungang Ology, Shanxi Province Key Laboratory of Microstructure Electromagnetic Functional Materials, and Institute of Solid State Physics, Shanxi Datong University, Datong 037009, China; zhangyw202110@163.com; 2 Yungang Academy, Datong 037007, China

**Keywords:** Yungang Grottoes, Cave 29, deterioration features of stone cultural relics, field investigation, multi-technique testing

## Abstract

The Yungang grottoes (5th to 6th centuries AD) were one of the largest and most artistically valuable ancient grotto complexes in China and were a precious World Cultural Heritage site. Influenced by the natural environment and human factors, various deterioration features appeared in the Yungang grottoes. Taking Cave 29 as the research object, field investigations were conducted to analyse the deterioration features and their distribution within Cave 29. To further analyse the formation mechanisms of deterioration features and the preservation conditions, multi-technique testing methods were used. Infrared thermal imager clearly identified hollowing, cracks and surface salt efflorescence zones. Leeb hardness testing on Cave 29’s walls gave average rebound values of 283–308 HLD, all values were lower than the average rebound values of fresh rock (661.4 HLD), indicating weathering-induced surface hardness reduction. Ultrasonic testing showed the east wall had the highest wave velocity (avg. 1928 m/s), indicating the best preservation, while the south wall had the lowest (avg. 1378 m/s), indicating the most severe weathering. Microscopic observations, ion chromatography measurements, and p-XRF were preliminarily determine that CaSO_4_ could be preliminary inferred as the dominant soluble salt phase present at this site. All these findings provided scientific data to support preventive protection and scientific conservation.

## 1. Introduction

**Figure 1:**
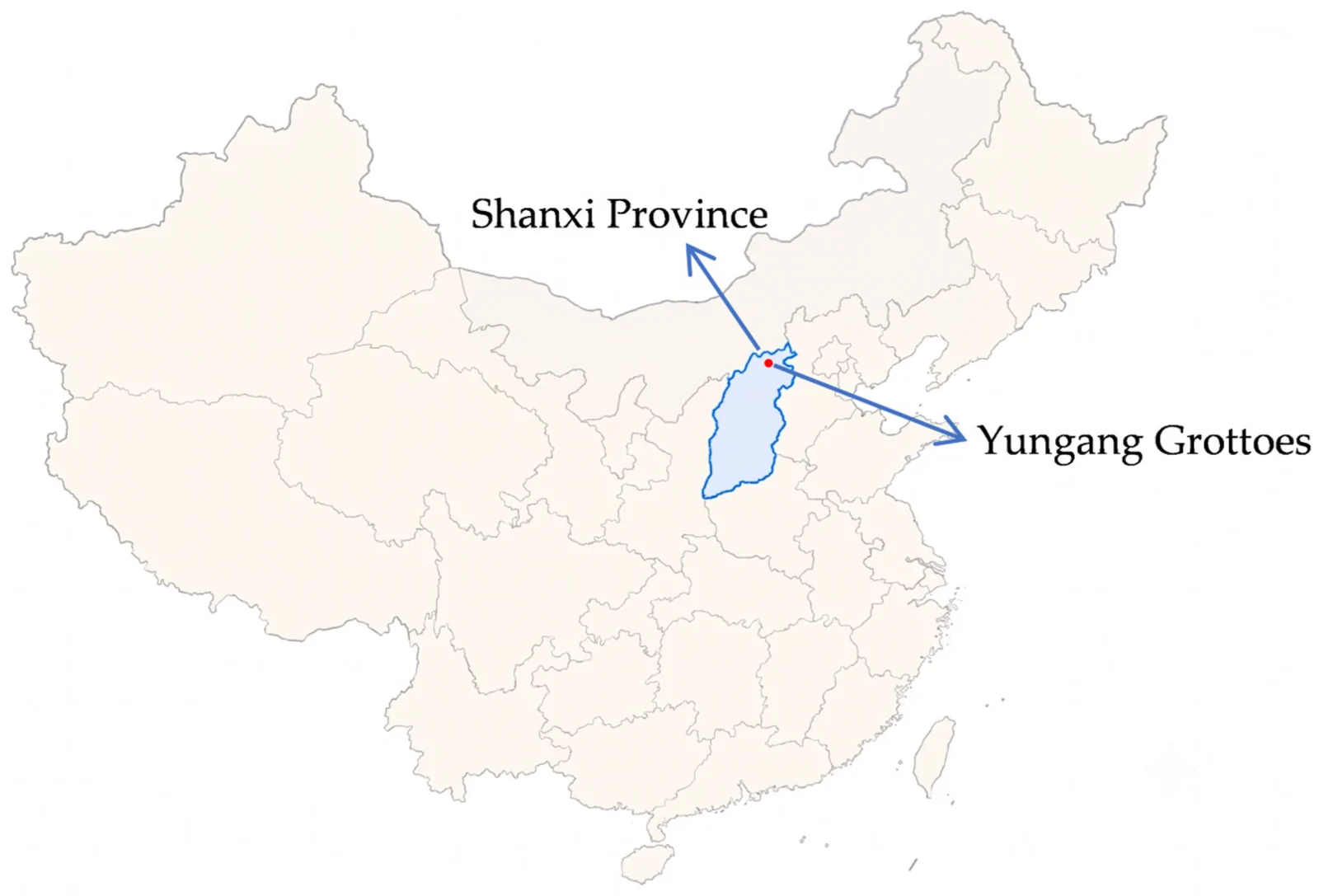
Geographical location map of Yungang Grottoes.

The Yungang Grottoes were situated on the southern slopes of Wuzhou Mountain in the western outskirts of Datong City, Shanxi Province, China (Figure 1). As the earliest large-scale grotto complex constructed east of Xinjiang in China, it mobilized and concentrated all the human, material, and financial resources of the Northern Wei imperial family at that time. It stood as a quintessential example of grotto excavation during the Northern Wei Dynasty, embodying substantial historical, artistic, and scientific significance [1]. As one of the largest ancient grotto complexes in Chinese history, the Yungang Grottoes was renowned alongside the Dunhuang Mogao Caves, the Longmen Grottoes, and the Maijishan Grottoes as the four great grottoes art treasures of China. The excavation of the Yungang Grottoes began in the first year of the Heping era (460 AD) during the reign of Emperor Wencheng of the Northern Wei Dynasty and continued until the fifth year of the Zhengguang era (524 AD) under Emperor Xiaoming, lasting a total of 64 years. Li Daoyuan, a renowned geographer of the Northern Wei Dynasty, described the spectacular scene during the excavation of the Yungang Grottoes at that time “Chiseled into the mountain rocks, with structures built against the cliffs, the Buddha’s appearance was lifelike, enormous, and magnificent—a rarity in this world [2].” This sentence was a true portrayal of the Yungang Grottoes. Their excavation was of epoch-making significance. They were the first to embed the idea that “the emperor was the Buddha,” and they combined caves with temple architecture to found the first imperial grotto temple, whose influence on later cave design and statuary was enduring. Constructed in the 5th century AD, the Yungang Grottoes possessed a history spanning 1500 years [3]. Owing to the impacts of various natural forces, environmental pollution, and human factors, the weathering process of the Yungang Grottoes accelerated, resulting in various deterioration features on the surfaces of the stone cultural relics [4]. It was urgently necessary to conduct a detailed deterioration feature investigation and multi-technique testing analysis on specific caves within the Yungang Grottoes, so as to provide scientific data for better cultural relic protection in the future.

Over the past two decades, the deterioration of sandstone heritage attracted increasing scholarly attention, with research efforts broadly falling into three thematic clusters. The first cluster concerned disease inventory and classification. Scholars used field investigation methods to investigate the weathering, water seepage, cracks and other deterioration features of stone cultural relics. Based on the current deterioration feature status of stone cultural relics, they provided relevant protection suggestions and achieved certain results [5,6,7,8]. At present, there was a growing array of methods available for performing such investigations, among them some being welcomed for analysis on the deterioration features of the Yungang Grottoes [9,10,11]. Liu et al. [12] conducted a comprehensive on-site survey of 45 caves at Yungang, identifying 12 distinct deterioration types and employing SEM, FT-IR, XRD, and EDAX to characterize their chemical and mineralogical compositions. While this work provided a valuable baseline, its primary focus was descriptive—cataloging symptoms rather than diagnosing the underlying physical mechanisms.

The second cluster addressed physicomechanical property characterization of stone cultural relics. Zhang et al. [13] measured compressive strength, effective porosity, and longitudinal wave velocity of Yungang sandstone samples, establishing empirical correlations among these parameters. Studies at other sandstone heritage sites, such as Petra [14] and Angkor [15], similarly demonstrated that mechanical properties varied significantly with moisture content and salt crystallization cycles, suggesting that site-specific environmental factors played a critical role in weathering progression.

The third and most methodologically relevant cluster involved multi-technique diagnostic techniques. Recent international studies increasingly advocated for combined approaches—for instance, integrating surface hardness measurements with ultrasonic velocity to capture different depth scales of property alteration [16,17]. In the Chinese context, Liu et al. [18] developed a laser-scanning-based protocol for hazardous rock-mass identification on steep slopes, demonstrating good applicability at Baifoshan. Notably, Hong et al. [19] recently introduced a quantitative assessment framework for sandstone weathering at Yungang, integrating multiple testing parameters into a composite index for overall weathering-grade classification. This represented a significant step toward standardized diagnostics. However, the integration approach inherently averaged out the discrepancies among individual parameters-discrepancies that might themselves carry important diagnostic information. Specifically, if surface hardness and internal wave velocity yielded conflicting signals, this discordance might indicate vertical heterogeneity in the weathering profile, rather than measurement error. This phenomenon—the decoupling between surface and subsurface properties—had been documented in weathered stone monuments elsewhere [20] but had not been systematically investigated at the Yungang Grottoes.

In situ multi-analytical characterization became a mainstream workflow for assessing the deterioration of porous sandstone heritage worldwide. Multi-technique geophysical techniques, including ultrasonic pulse velocity and Leeb hardness testing, were widely deployed to evaluate rock integrity, mechanical degradation and concealed subsurface defects without sampling. Nevertheless, such geophysical methods alone could hardly decode chemical weathering and salt-related damage; hence complementary compositional approaches were frequently integrated. Portable X-ray fluorescence (p-XRF) provided rapid in situ elemental screening for surface alteration layers, while minimally invasive powder sampling enabled further laboratory identification: ion chromatography quantified soluble anions, optical and digital microscopy visualized salt-crystal micromorphology. Some studies built quantitative weathering-evaluation frameworks based on multi-source datasets across the whole grotto zone. However, fewer efforts concentrated on individual representative caves, where surface salt-crystallization hazards and internal rock-mass deterioration were jointly interpreted to reveal site-specific degradation mechanisms. This gap motivated the present combined non-destructive and minimally invasive analytical investigation of Cave 29.

This study focused on Cave 29 of the Yungang Grottoes as its research subject, utilizing field investigation methods and multi-technique testing techniques to assess the deterioration feature present in the cave. The deterioration feature investigation of Cave 29 in the Yungang Grottoes primarily employed methods including cameras, steel rulers, color charts, and deterioration feature survey forms to document data on various deterioration features, such as hollowing, weathering, cracks, and surface salt efflorescence. Concurrently, infrared thermal imager, ultrasonic pulse velocity, Leeb hardness, portable X-ray fluorescence spectroscopy (p-XRF), ultra-deep field microscope, ion chromatography were employed for the multi-technique analysis of deterioration feature conditions. The findings revealed the existence of seven distinct types of deterioration features. This study primarily conducted deterioration feature investigations and multi-technique analysis on four types of deterioration features: hollowing, weathering, cracks, and surface salt efflorescence. It examined the spatial distribution, chemical composition, and preservation conditions of the four deterioration features, delved into the formation mechanisms underlying the deterioration features in stone cultural relics, and explored effective approaches for quantitatively characterizing these deterioration features through multi-technique testing techniques. This research held substantial reference value for the study of deterioration features in stone cultural relics.

## 2. Experimental Methods

### 2.1. Cave Information

**Figure 2:**
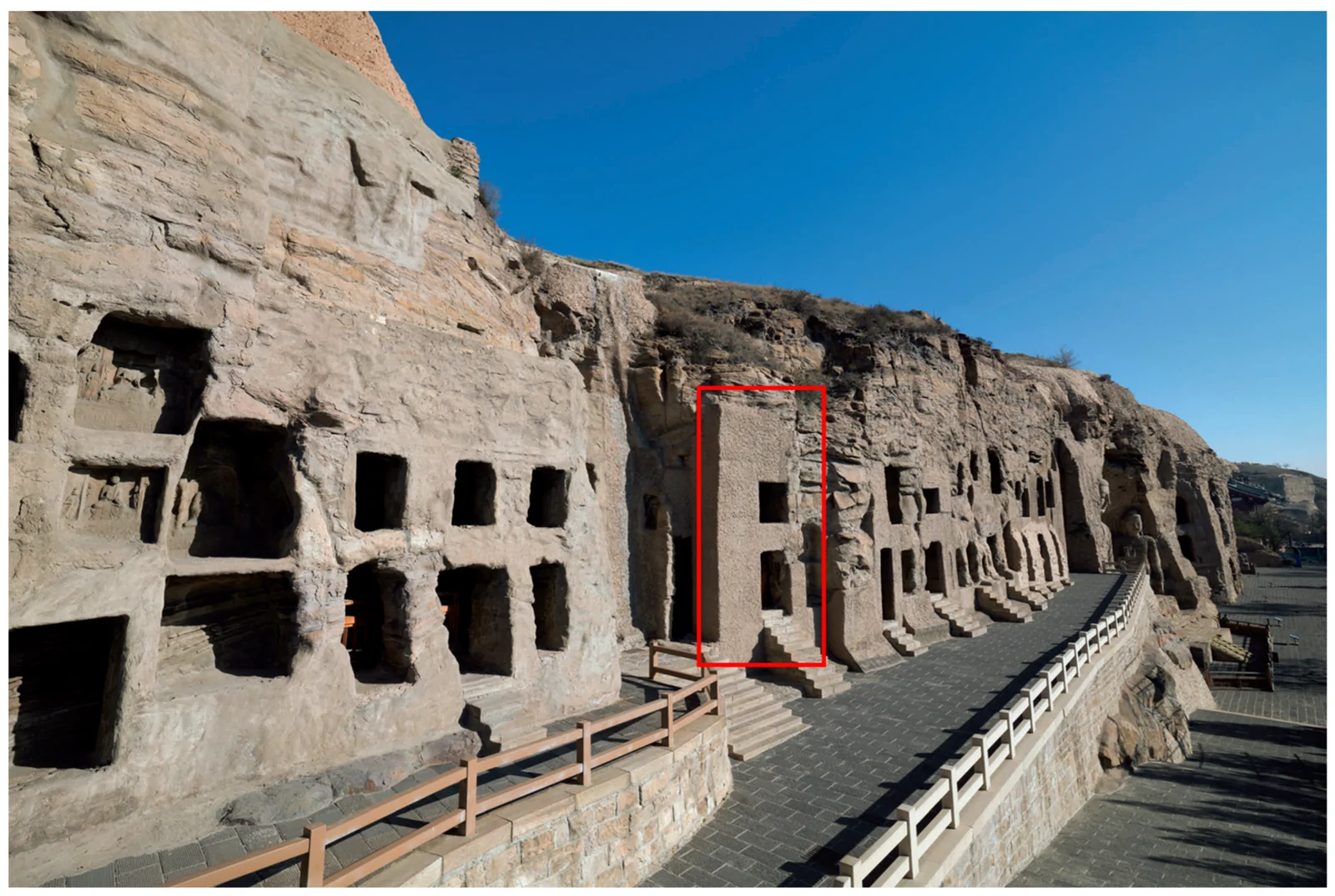
Cave 29 in the western cave cluster [21].

Cave 29 was situated in the middle section of the western cave cluster within the Yungang Grottoes (Figure 2) [21], spanning from cave 14 to cave 45 and encompassing a total of 32 extant major caves [22]. Centered on the earliest five caves of Tanyao, the caves located to the west of cave 20 were all constructed during the late Northern Wei Dynasty, subsequent to Emperor Xiaowen’s relocation of the capital to Luoyang. Owing to the absence of a national-scale engineering endeavor and unified planning, caves 21 to 45 did not consist of grouped caves. Instead, they were all small to medium-sized niches characterized by diverse layouts and orderly structures. Judging from the plan and elevation drawings, Cave 29 stood as the sole cave among the western grotto niches that faced southwestward.

**Figure 3:**
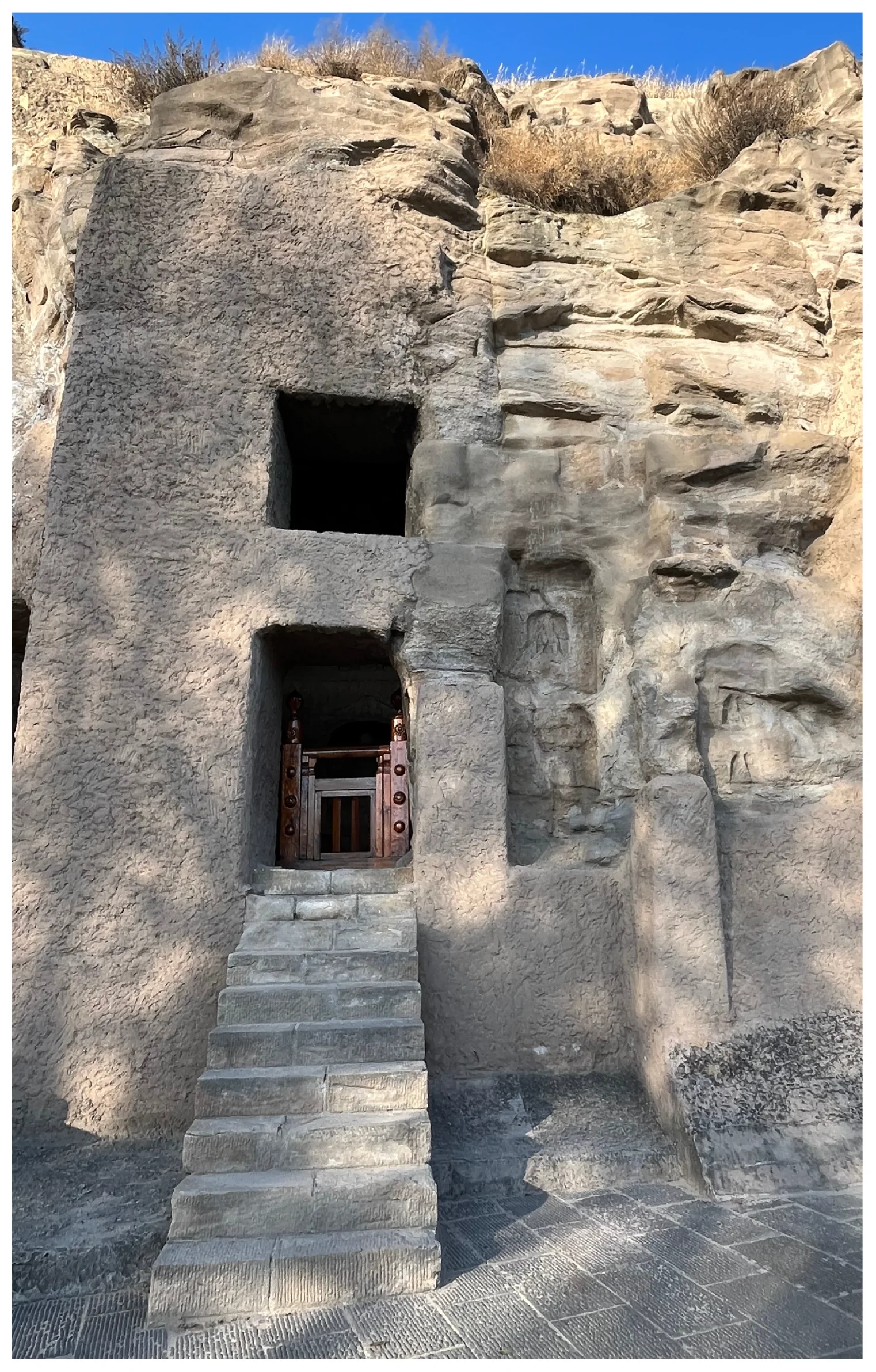
Photo of the exterior view of Cave 29.

The majority of the external facade of Cave 29 (Figure 3) had collapsed, and it had undergone substantial reinforcement and restoration using modern cement materials. A cave door was carved in the center, and a bright window was opened directly above the cave door. The cave was rectangular, with a width of 4.15 m, a depth of 3.2 m, and a height of 4.5 m. The north and east walls of the grotto featured two tiers of niches, yet the carvings originally present on the niche bases were no longer extant. A cave entrance was constructed beneath the southern wall, accompanied by a clear window positioned above it. The western portion had suffered complete collapse, whereas the eastern section remained relatively intact and was structured into two tiers. The west wall featured multiple tiers of niches, with the southern section having collapsed as a result of the south wall’s collapse. Nevertheless, the configurations of the niches remained discernible.

### 2.2. Infrared Thermal Imager

The employment of the HIKMICRO OQ35 infrared thermal monocular (Hangzhou HIKMICRO Sensing Technology Co., Ltd., Hangzhou, China) facilitated the identification of deterioration features, including hollowing, surface salt efflorescence, and cracks in Cave 29. This approach enabled an intuitive, precise, and swift quantitative characterization of these deterioration features, along with an analysis of their formation mechanisms. The infrared thermal monocular incorporated a highly sensitive VOx sensor and a 35 mm large-aperture lens, achieving a thermal sensitivity superior to 35 mK. The detector boasted a resolution of 640 × 512 pixels, with a pixel size of 17 μm and a lens focal length of 35 mm, and a maximum detection range of 1235 m. The device supported maximum temperature point tracking and distance measurement. The ambient temperature remained stable throughout the tests, and its temperature measurement accuracy reached ±1 °C or ±1%. Except for the staff operating the infrared thermal imager, other personnel were prohibited from entering Cave 29. The instrument supported FFC (Flat Field Correction) and DPC (Dead Pixel Correction) to optimize image quality. Images were captured directly at deterioration feature locations within Cave 29. More than three sets of infrared images were repeatedly acquired for each deteriorated area, and valid data were selected after removing abnormal frames. Images could be exported to a computer using a data cable or transmitted wirelessly via an App.

### 2.3. Ultrasonic Pulse Velocity

The Proceq Pundit PL-200 ultrasonic pulse velocity tester (Proceq SA, Zurich, Switzerland) was utilized to assess weathering and hollowing on four walls and the ceiling of Cave 29, aiming to assess the weathering degree of rock materials in stone relic inspection. The fundamental principle was that ultrasonic pulses were emitted toward the target, followed by analysis of the reflected signals. Changes in parameters including wave velocity, waveform and attenuation could be measured to deduce the internal physical and structural characteristics of materials. The ultrasonic method showed extensive application potential and popularization value in identifying weathering grades of stone cultural relics and assessing rock reinforcement performance.

Ultrasonic waves propagated far more slowly in liquid and air than in solid media. Accordingly, denser rock masses yielded higher ultrasonic wave velocities, while rock masses with more defects exhibited lower wave velocities. Representative regions within Cave 29 were chosen to conduct measurements on the four walls and the ceiling. For measurements taken on different walls using ultrasonic instruments, the ceiling was measured 9 points, while the other four walls were each measured 10 points. Using the surface indirect transmission method, the transmitting transducer (T) and receiving transducer (R) were positioned on the same testing surface and maintained a fixed separation of 0.6 m. The instrument is equipped with two transducers with a rated frequency of 54 kHz, capable of conforming to rough or curved surfaces without the need for coupling gel. The measurement technique utilized is based on ultrasonic pulse velocity, with the pulse voltage configured at 100 V and the receiver gain adjusted to 34 db. Prior to testing, the instrument was calibrated using a standard block of known ultrasonic velocity to ensure measurement accuracy. The transducers were maintained perpendicular to the rock face with uniform pressure so as to eliminate artificial errors.

Given the substantial area of weathering and pulverizing wall surfaces within the grotto, couplant was omitted during this data collection process to prevent the emergence of new damage stemming from couplant adhesion to these surfaces. In this test, point-contact sensors were employed, which exhibited a relatively lower accuracy compared to area sensors. To ensure the precision of the measured values, ten tests were conducted at each sampling point, and the mean value was subsequently calculated. During the testing of rock walls at Cave 29 of the Yungang Grottoes, only one outer surface was available for operation. Since the direct transmission method demanded testing from two opposite sides, this technique could not be realized on site for Cave 29, which only possessed a single exposed surface. Limited by wall surface conditions, the direct transmission method could not be employed. Instead, the indirect transmission method was adopted, where the transmitting and receiving transducers were positioned on the same surface of the specimen, and the signal amplitude decreased accordingly.

At each grid point, ten consecutive ultrasonic pulse velocity measurements were recorded, and the average value was calculated for that point. For each wall, the overall mean value, standard deviation (SD), and data range (minimum–maximum) were calculated from all valid grid points. The SD reflects the inter-point variability within each wall, which is considered a meaningful indicator of spatial heterogeneity. The number of independent measurement points (*n*) for each wall is also reported.

### 2.4. Leeb Hardness Tester

Hardness measurements with grid point layout were carried out on different wall surfaces of Cave 29 using the Proceq Equotip Live Leeb D Leeb hardness tester (Proceq SA, Schwerzenbach, Switzerland). It was a scientific, efficient, relic-friendly testing technique. Systematic point arrangement and rigorous operations provided critical data for the deterioration feature assessment and conservation of the grotto. The instrument worked by driving an impact body to strike the rock surface, and hardness values (HLD) were calculated according to the ratio of rebound velocity to impact velocity. Harder rocks with better elasticity yielded higher rebound velocities and higher HLD values. Conversely, weathering or loose rocks absorbed more energy, which led to lower HLD values. The Leeb hardness tester delivered low impact energy and produced an indentation of merely approximately 0.5 mm, so it was highly suitable for fragile cultural relics. A single measurement took only 2 s, which facilitated dense point layout on the wall surfaces of Cave 29. Therefore, it represented a portable and efficient testing approach. This Leeb hardness tester necessitates the utilization of an iOS device, with a measurement scope spanning from 100 to 1000 HLD, a test force of 11 Nmm, and a measurement accuracy of ±4 HLD (0.5% at 800 HLD).

Before testing, calibration using the instrument’s built-in verification tool was suggested to ensure data accuracy. A systematic grid layout of measuring points with a spacing of 0.6 m was implemented to fully investigate the surface hardness of the walls in Cave 29 of the Yungang Grottoes. Several severe deterioration feature areas were avoided when arranging points, this might introduce potential deviations from the reported average values. Nine measurements were taken at varied positions surrounding each measuring point, after which invalid readings were removed.

### 2.5. p-XRF

The DPO-6000+ p-XRF (Olympus Scientific Solutions Americas, Waltham, MA, USA) was employed for qualitative and quantitative analysis of chemical elemental compositions of stone cultural relics and their weathering products, which could quickly obtain the elemental composition of the cultural relics. An X-ray tube with a silver (Ag) target X-ray tube and a silicon drift detector were utilized. The excitation voltage spanned from 8 to 40 kV, and the electric current ranged between 5 and 200 μA. The instrument was calibrated based on the fundamental parameter method and possessed an automatic calibration function. After power-on, the instrument was preheated for 15–30 min to stabilize its internal temperature. Before measurement, the equipment status was verified using the calibration block provided by the instrument. As the Yungang Grottoes were a World Cultural Heritage site, the principle of non-destructive testing had to be followed, and no chemical or physical treatment was conducted on Cave 29. Measurements were carried out at positions with flat surfaces that fully covered the detection window. The single measurement duration was set to 120 s, and the beam spot diameter was 10 mm. Elemental analysis was performed on different wall surfaces of Cave 29. Measuring points were arranged in a grid pattern at an interval of 0.6 m. Three tests were implemented at each point, and the average value was calculated from the obtained results.

### 2.6. Ultra-Deep Field Microscope

The Hirox HRX-01 ultra-depth field microscope (Hirox Co., Ltd., Tokyo, Japan) was adopted to acquire two-dimensional or three-dimensional microscopic images of cultural relics. The microscope was equipped with a CMOS image sensor with effective pixels of 2448 (H) × 2048 (V) and supported 4K ultra-high-definition resolution. It automatically captured images layer by layer within a set range and achieved real-time dynamic acquisition at 50 frames per second. The HRX-01 was fitted with multiple sets of motorized zoom lenses. A wide-angle high-resolution telecentric zoom lens, offering a magnification ranging from 20× to 200×, was mainly used. It was suitable for general surveys of large-area surface morphology and observations of crack and pore distribution. The microscope was equipped with a high-brightness, long-life LED light source with a colour temperature of 5700 K. Trace samples collected from concealed locations were placed on glass slides for observation, and the maximum resolution reached 0.05 μm. The HRX-01 was equipped with a high-precision motorized *Z*-axis. Either the auto-focus function of the instrument or manual fine-tuning was utilized to ensure clear imaging. After focusing and light source adjustment were completed, high-quality single-frame images were recorded with one click via the capture button on the software interface or the physical key on the device. Images captured by the microscope could be exported in various common formats such as TIFF, BMP and JPEG. The above imaging conditions and acquisition strategies guaranteed the clarity, representativeness and repeatability of the observation results.

### 2.7. Ion Chromatography

The Metrohm ECO IC (Metrohm AG, Herisau, Switzerland) ion chromatograph was employed to analyze surface salt efflorescence in Cave 29. The core purpose of ion chromatography was to quantitatively analyse the species and contents of soluble salts in rock samples. The key step of such analysis involved extracting salts from solid samples into aqueous solutions for subsequent chromatographic testing. This instrument was primarily equipped with anion and cation chromatography columns and an 863 compact autosampler automatic sampler. In the surface salt efflorescence area on the east wall of Cave 29, approximately 5 g of rock powder was scraped from a depth of 2–3 cm using sterile scalpels or small drill bits. This sampling approach was widely adopted for surface salt efflorescence analysis of stone cultural relics. The collected samples were immediately placed in clean, sealed sample bags or centrifuge tubes, labelled with corresponding location information to avoid cross-contamination. A certain mass of rock powder (e.g., 1.0 g) was accurately weighed and recorded. The powder was transferred into centrifuge tubes, and 10–20 mL of ultrapure water (resistivity ≥ 18.2 MΩ·cm) was added. The mixture was shaken or treated ultrasonically for 15–30 min to achieve full dissolution of soluble salts. The extracts were centrifuged at 4000–5000 rpm for 10 min, or filtered through 0.22 μm microporous membranes, and the supernatant was retained for testing. After prepared samples were loaded onto the 863 compact autosampler, anion contents were analysed. The measurement results reflected the anion concentrations related to surface salt efflorescence on the east wall of Cave 29. The typical detection range was 0.2–200 mg/L. The flow rate was usually set between 0.7 and 1.0 mL/minutes, and the injection volume ranged from 10 to 20 μL. The typical analysis duration for a single sample was 18–30 min.

## 3. Results and Discussion

**Figure 4:**
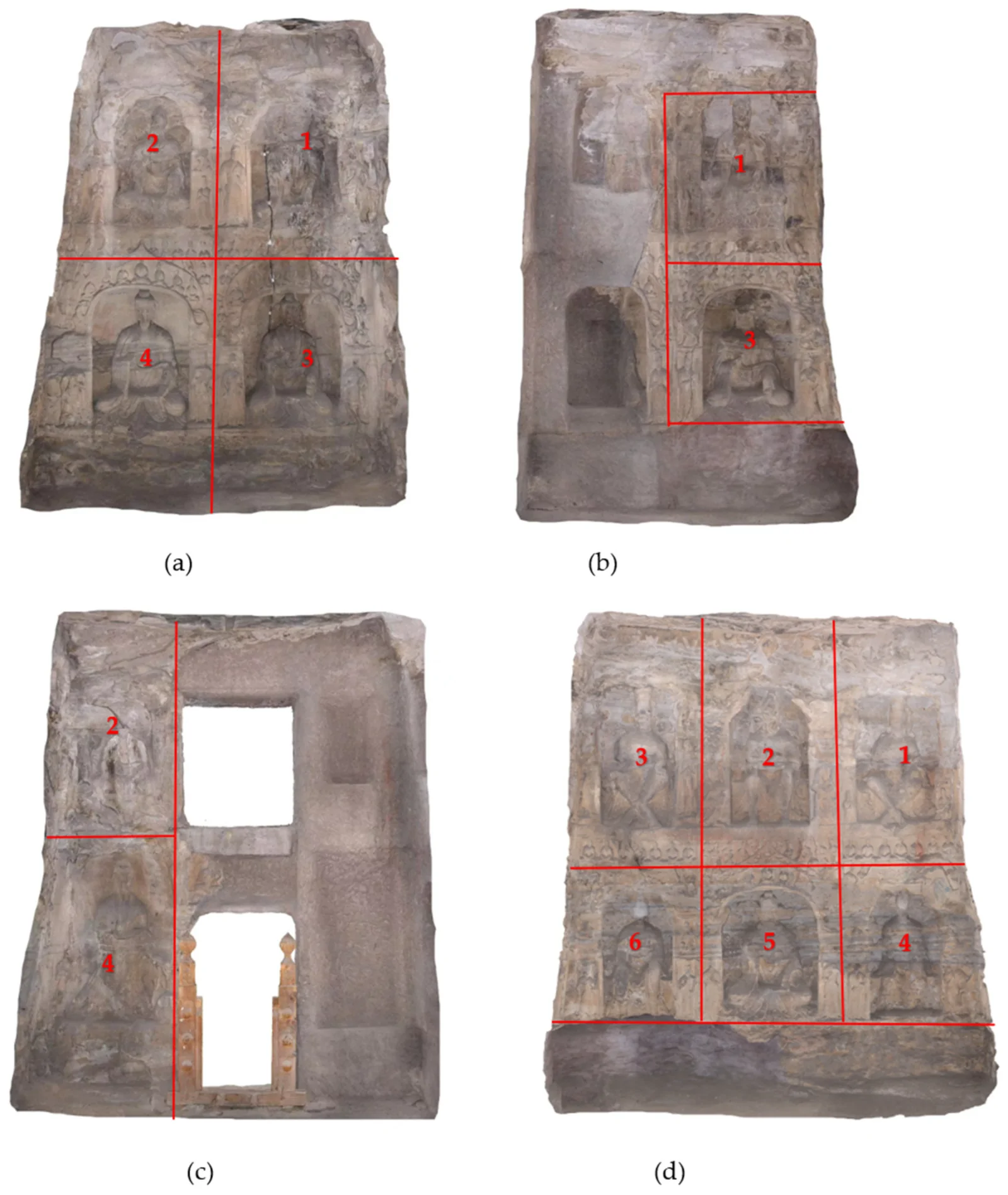
(**a**) Zones 1–4 in the east wall. (**b**) Zones 1 and 3 in the west wall. (**c**) Zones 2 and 4 in the south wall. (**d**) Zones 1–6 in the north wall.

This study employed field investigation methods to conduct an on-site investigation of Cave 29 at the Yungang Grottoes, revealing that the predominant existing deterioration features in the cave include weathering, hollowing, cracks, surface salt efflorescence, exfoliation, surface contamination, and anthropogenic graffiti. In addition, exfoliation, surface contamination, and anthropogenic graffiti were observable with the naked eye and were therefore not subjected to further instrumental investigation. The field investigation of stone cultural relics was carried out targeting the cave wall surfaces, following an investigation sequence from left to right and top to bottom. The statues within the niches of Cave 29 were adopted as the zoning criterion, as shown in Figure 4. The east wall was divided into Zones 1–4 (Figure 4a), and the north wall was divided into Zones 1–6 (Figure 4d). Owing to half coverage by cement layers on the west and south walls, only Zones 1 and 3 were measured on the west wall (Figure 4b), while merely Zones 2 and 4 were tested on the south wall (Figure 4c). Multi-technique testing techniques such as infrared thermal imager, ultrasonic pulse velocity, Leeb hardness, p-XRF, ultra-deep field microscope, ion chromatography were employed to analyze the hollowing, weathering, cracks, and surface salt efflorescence in Cave 29 of the Yungang Grottoes, aiming to explore the formation mechanism of cultural relic deterioration features.

### 3.1. Hollowing

Hollowing denoted the phenomenon in which the surface layer of stone cultural relics swelled and detached to form cavities, yet without undergoing complete exfoliation [23]. Through investigation of Cave 29 of the Yungang Grottoes, it was found that the area of the hollowing section was 3.31 square meters. There were 18 hollow spots on the ceiling, 2 on the north wall, 7 on the east wall, 1 on the south wall, and 3 on the west wall. Based on the deterioration feature map, the hollowing section on the ceiling exhibited the largest area, measuring 2.772 square meters. This specific area constituted 83.75% of the total hollowing area within Cave 29 and 20.87% of the entire ceiling area of Cave 29.

**Figure 5:**
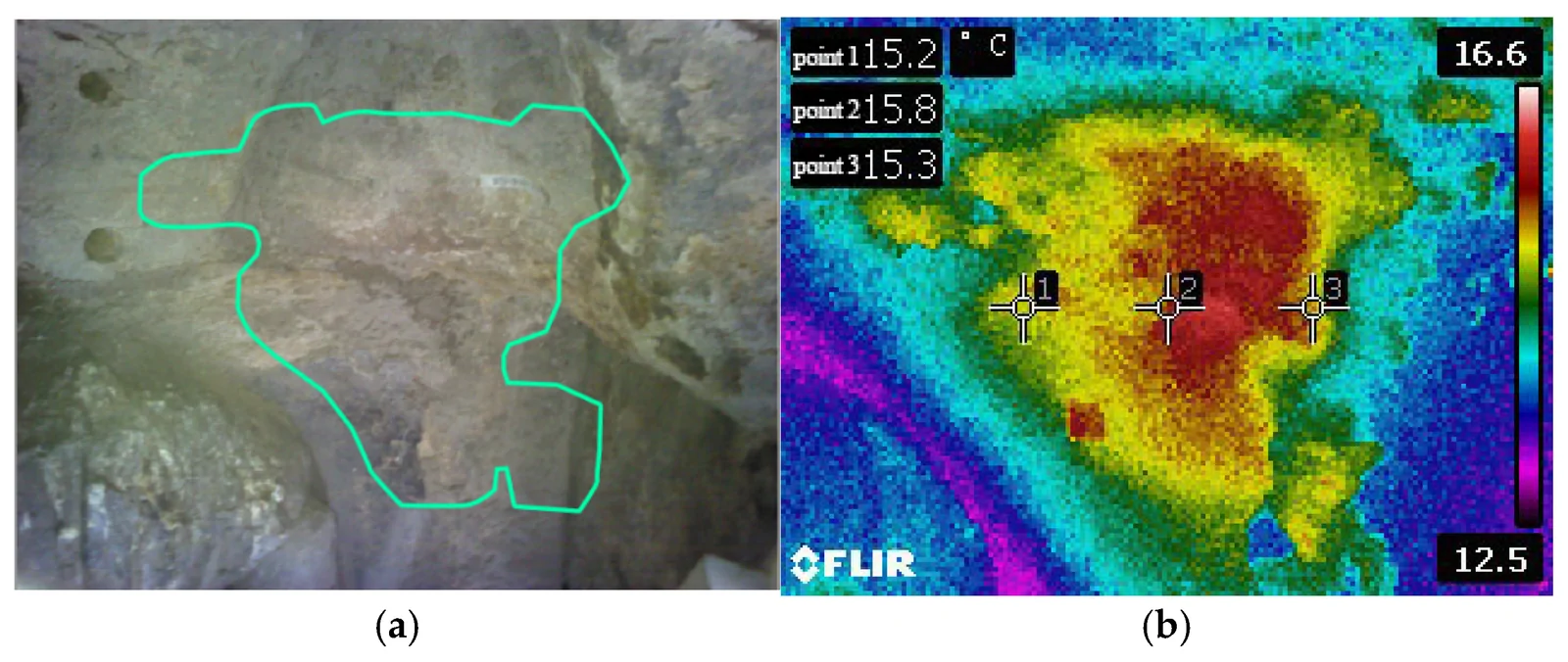
(**a**) The hollowing area of the left niche on the east wall in Cave 29. (**b**) Infrared thermal imager diagram in hollowing area of the left niche on the east wall. Figure 5a was photographed by Miao Tian of Yungang Academy.

**Figure 6:**
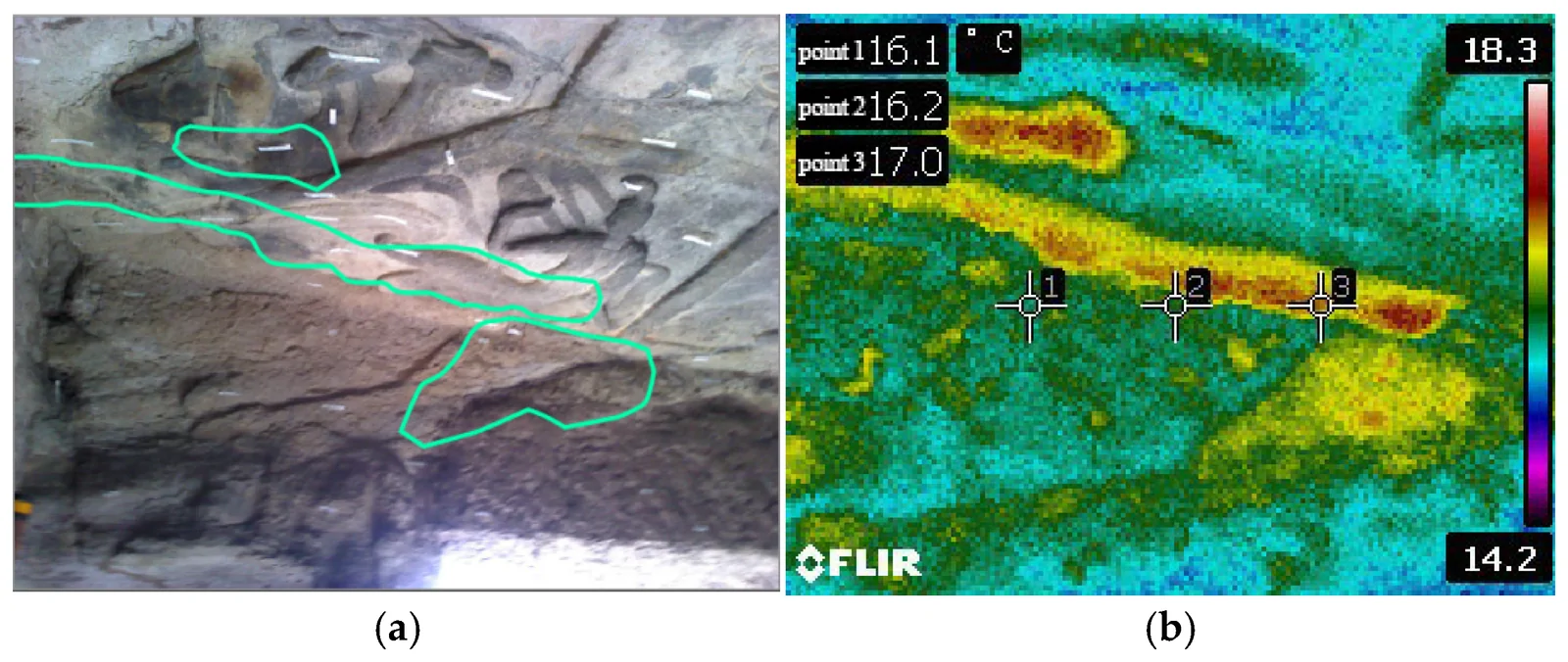
(**a**) The hollowing area on the southeast side of the ceiling in Cave 29. (**b**) Infrared thermal imager diagram in hollowing area on the southeast side of the ceiling. Figure 6a was photographed by Miao Tian of Yungang Academy.

To address the hollowing observed on the ceiling and east wall of Cave 29 in the Yungang Grottoes, infrared thermal imager was employed for detailed identification (Figure 5 and Figure 6). The thermal images (Figure 5b and Figure 6b) clearly delineated the hollowing areas, with red and yellow high-temperature bands indicating higher temperatures than the surrounding intact medium. To verify these results, a resonance echo detection rod and manual tapping inspection were used. The tapping tests showed that the hollowing positions largely overlapped with the high-temperature regions, and the crisper sound produced in those areas suggested greater hollowing depth. Overall, the manual tapping results correlated highly with the infrared data, confirming that infrared thermal imaging can serve as a complementary method to manual tapping.

If there was hollowing in stone cultural relics, it induced discontinuity within the rock mass, giving rise to a relatively thermally insulating air layer, which caused a notable disruption in heat conduction. Concurrently, the temperature within the mountain remained relatively stable, acting as a buffer to mitigate external temperature fluctuations. For rock masses with hollowing, the surface layer underwent freezing due to low nighttime temperatures, while the internal heat of the mountain struggled to compensate for the surface layer. Consequently, the temperature of the rock mass influenced by the hollowing was notably lower than that of the surrounding continuous rock mass. During the daytime, solar radiation elevated the surface temperature of the rock mass. The heat within rock masses containing hollowing was poorly conducted into the mountain’s interior, resulting in heat accumulation on the cliff surface and consequently causing the temperatures of these hollowing rock masses to be higher than those of the surrounding areas.

### 3.2. Weathering

**Figure 7:**
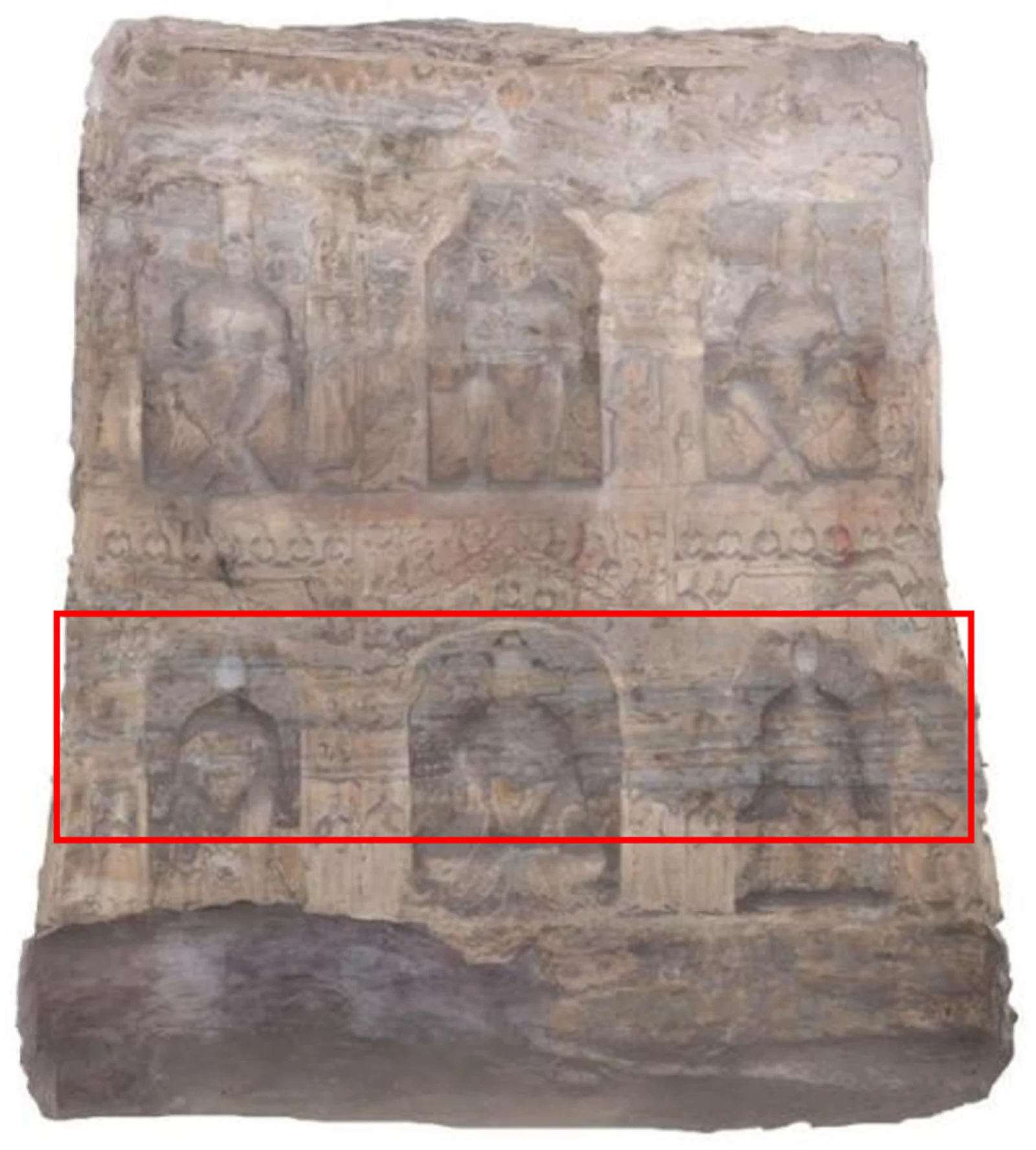
The pronounced weathering zone on the north wall of Cave 29.

Weathering referred to the surface deterioration feature of stone cultural relics resulting from the destructive influence of external natural factors. The ceiling and the western wall of Cave 29 at the Yungang Grottoes remained in a well-preserved state, exhibiting no evidence of weathering. A pronounced weathering zone (Figure 7) was present on the head and upper torso sections of the Buddha niche located on the lower layer of the north wall within the cave, which was quite severely weathering. On the south wall of the cave, there were two groove-shaped weathering and two hole-shaped weathering. The east wall of the cave exhibited a total of seven weathering features, comprising five hole-shaped weathering and two groove-shaped weathering.

During this inspection, a p-XRF analyzer was employed to perform qualitative and quantitative analyses on the chemical element composition of both stone cultural relics and their weathering products in Cave 29. Based on the analysis of p-XRF detection data collected from selected sampling points, apart from silicon, Cave 29 also exhibited significantly high contents of calcium, aluminum, and sulfur. The cement was mainly composed of calcium compounds, with its fundamental constituents being SiO_2_, Al_2_O_3_, and CaSO_4_. According to the analysis of the inspection results, all wall surfaces in Cave 29 had undergone varying degrees of weathering, with distinct weathering grooves evident in zones 3 and 4 of the east wall, as well as zones 3, 4, and 5 of the north wall.

Through examination with a Leeb hardness, it was identified that the weathering zones on stone cultural relics of Cave 29 exhibited elevated porosity, consequently leading to a reduction in both strength and hardness. A Leeb hardness tester was employed to conduct grid-point hardness tests on four walls and the ceiling of Cave 29, with the testing scope encompassing all the areas affected by deterioration features identified in this investigation. In adherence to the principle of ensuring the utmost protection for cultural relics, we had deliberately circumvented areas suffering from severe pulverization or those unsuitable for testing purposes. At each designated measuring point, nine repeated measurements were performed, and the mean value of the nine data points was computed. It should be pointed out that since half of the west and south walls of Cave 29 were covered with cement, hardness testing was not undertaken on those covered regions.

Table 1 presented the average Leeb hardness values of five wall surfaces in Cave 29 of the Yungang Grottoes. Leeb hardness reflected the ability of rock surfaces to resist impact deformation. A higher Leeb hardness value indicated harder rock surfaces and a lower degree of weathering and loosening of the surface rock mass. A lower Leeb hardness value suggested more severe weathering, pulverization of surface sandstone, accompanied by significant degradation of surface mechanical properties. According to the average Leeb hardness values of the five wall surfaces summarized in Table 1, the regional hardness mean values followed the order: ceiling, south wall, west wall, east wall and north wall.

The weathering degree of stone cultural relics was assessed by utilizing the ratio of the mean hardness value of weathering zones to that of fresh rock specimens. The results (Table 1) indicated that the hardness and uniaxial compressive strength of the entire grotto were generally low. Specifically, the average Leeb hardness at the measurement points within the grotto spanned from 163 to 407 HLD. The average rebound hardness values on the wall surfaces of Cave 29 ranged from 283 to 308 HLD, all of which were significantly lower than the average rebound hardness values of fresh rock (661.4 HLD) [24]. This observation suggested that weathering processes had led to a reduction in the hardness of the rock surface. The fresh-rock reference ultrasonic pulse velocity values were obtained from in situ measurements on the outer wall surface on the west side of the gully between Caves 4 and 5 at the Yungang Grottoes [24]. This location was composed of medium-grained lithic sandstone, which was lithologically consistent with the host rock of Cave 29. The reference measurements were conducted under dry, sunny, and windless conditions on the outer cliff face. By contrast, the ultrasonic pulse velocity measurements inside Cave 29 were carried out under in situ ambient moisture conditions, owing to the semi-enclosed environment of the cave and the absence of active drying measures. The same equipment (with a fixed transducer spacing) was used for both the reference and in-cave measurements to minimize configuration-induced variability. It should be noted that the moisture conditions between the two measurement settings were not strictly identical, which might affect the direct comparability of the derived classification thresholds. Therefore, the weathering grades assigned to the cave walls based on these thresholds were interpreted as semi-quantitative estimates rather than absolute determinations, and the relative ranking among the walls was emphasized over the absolute grade values.

**Figure 8:**
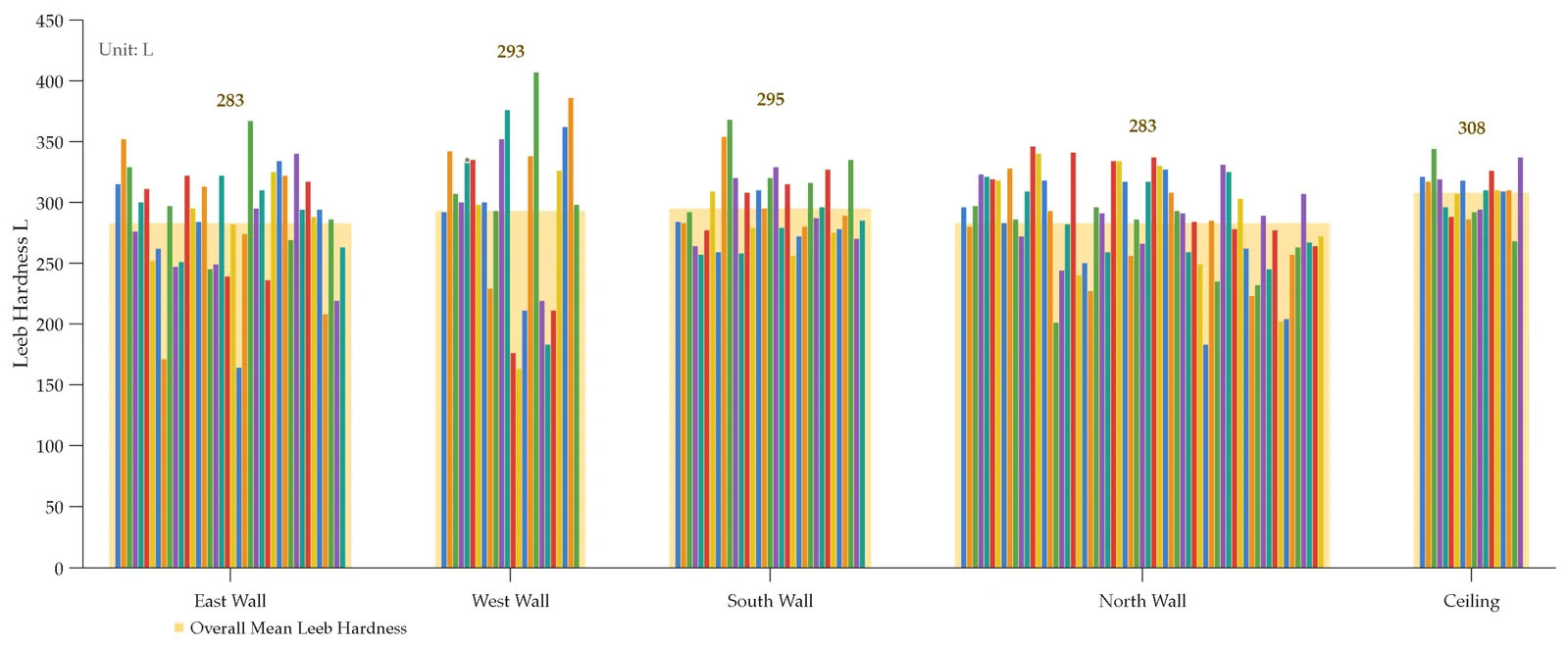
The average Leeb hardness value of the measurement points on the wall of Cave 29.

Based on the average Leeb hardness values obtained from the five wall surfaces, further investigation was conducted into the fluctuation discrepancies among individual measuring points within each wall surface and the localized heterogeneity of weathering. The average Leeb hardness values of the measuring points on the various wall surfaces of Cave 29 were plotted in Figure 8. The abscissa denoted the five wall-surface detection zones, while the ordinate denoted the Leeb hardness (L). For each wall surface, a series of colored column chart were plotted, each representing the averaged Leeb hardness value derived from nine repeated measurements at a single measuring point. The values annotated with yellow numerals in the figure corresponded to the mean Leeb hardness values of the respective wall surfaces listed in Table 1, which signified the overall average Leeb hardness levels across the five different wall surfaces.

The average Leeb hardness of the ceiling was 308 HLD, the highest value among the five wall surfaces of Cave 29. Its hardness ranged from 268 HLD to 344 HLD. Most measuring points on the ceiling showed relatively high hardness values, yet several obvious low-value points were detected. This phenomenon illustrated that the ceiling had high overall hardness, accompanied by local weathering and loosening areas. Half of the west wall was covered by cement for cultural heritage protection, so only zones 1 and 3 were tested. Its Leeb hardness ranged from 163 HLD to 407 HLD, with an average value of 293 HLD. The hardness distribution was uniform in the upper zone 1, while the lower zone 3 showed drastic fluctuation, corresponding to the minimum and maximum hardness values of the west wall. The east wall had an average Leeb hardness of 283 HLD, the lowest level inside Cave 29. Its hardness varied between 164 HLD and 367 HLD, and the hardness rose as the measuring height increased. Numerous measuring points concentrated in low-hardness intervals, revealing severe surface deterioration caused by weathering. Half of the south wall was also covered with cement, and only zones 2 and 4 were tested. Its Leeb hardness ranged from 256 HLD to 368 HLD, with a mean value of 295 HLD. Hardness declined gradually with the increase in height, and most measuring points in the central tested areas exceeded 280 HLD. The hardness data of the south wall distributed widely and stayed at an intermediate level among all wall surfaces. The north wall shared the same average Leeb hardness of 283 HLD as the east wall. Its hardness ranged from 183 HLD to 346 HLD, and its hardness increased with height. The middle and lower sections maintained low hardness values, most of which were below 300 HLD; the minimum value of 183 HLD originated from a typical weathering zone in the middle-lower north wall. Dense low-hardness measuring points also indicated prominent weathering-induced surface weakening on the north wall.

In summary, the ceiling had the highest surface hardness and the best surface integrity, followed by the south and west walls. A Leeb hardness value of 407 HLD was recorded on the west wall, which was directly associated with its good preservation. The east and north walls had the lowest hardness, where surface sandstone weathering was most significant. The east and north walls of Cave 29 demonstrate the minimum hardness values, attributable to the extensive flaky and powdery weathering observed on their surfaces. Based on on-site observations, these two walls represent areas with a relatively complex array of deterioration features. Notably, the north wall can be regarded as undergoing rapid weathering. The north wall has undergone relatively extensive weathering, primarily manifesting as powder-form and flake-form exfoliation, with a relatively loose surface layer and a low rebound value. All measuring points on the wall surfaces showed significant high-low fluctuations, proving that surface weathering was unevenly developed across Cave 29. Even the ceiling, with the highest average value, had localized low-value points, indicating that no completely uniform area existed. The overall data showed that weathering had significantly reduced the rock surface hardness of Cave 29.

Ultrasonic testing was performed on the various wall surfaces of Cave 29 by means of the area scanning technique. At each designated point, ten repeated measurements were conducted, and the mean value was subsequently calculated. For each wall, we had calculated the standard deviation (SD) based on the variability among all effective measurement points, along with the number of independent points (*n*) and the full data range (minimum–maximum). Table 2 presented the mean ± standard deviation (SD), the number of effective measurement points (*n*), and the minimum–maximum range for each wall. Based on the ranking of the velocity values, the east wall yielded the highest wave velocity, with a mean of 1928 m/s, suggesting that the east wall contained relatively few internal fractures and had undergone the least weathering, thus representing the area with the best preservation conditions within the cave. The south wall, in contrast, produced the lowest wave velocity, averaging 1378 m/s, which was the minimum among all measurement zones, indicating that weathering deterioration feature on the south wall was the most pronounced and that this area should be prioritized in future reinforcement and conservation efforts. The west wall and north wall exhibited wave velocities at an intermediate level relative to the other surfaces. The wave velocity of the ceiling was generally low, a finding that was closely associated with the extensive hollowing zones present on the ceiling.

**Figure 9:**
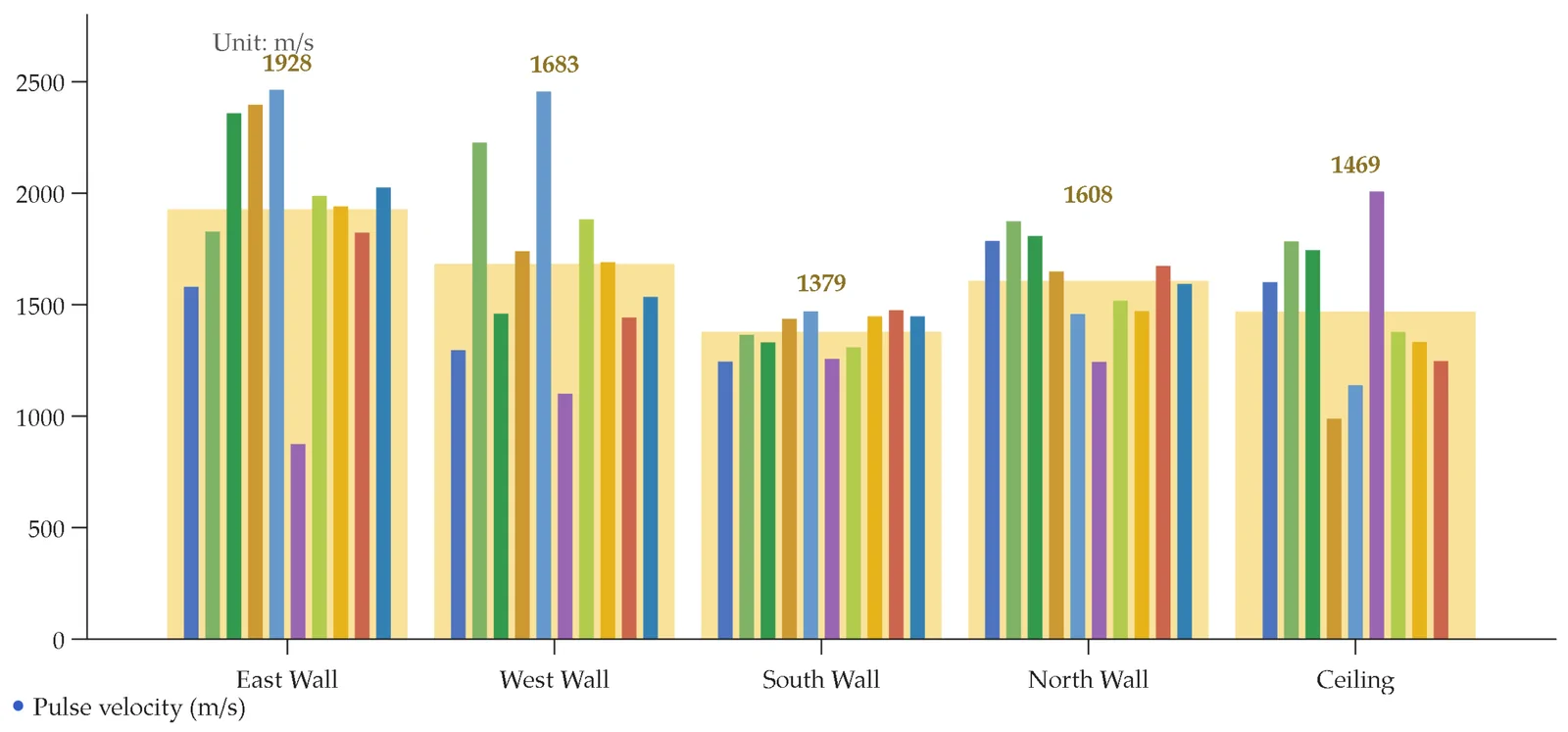
The average ultrasonic pulse velocity measurements of Cave 29.

Figure 9 presented the colored column chart of average ultrasonic pulse velocity data obtained from Cave 29 of the Yungang Grottoes. The abscissa denoted the five detection zones of Cave 29, namely east wall, west wall, south wall, north wall, and ceiling, while the ordinate denoted the wave velocity. For Figure 9, each colored column chart represented a specific measurement point on a wall. Taking the east wall as an example, the 10 colored column charts indicated that 10 points were measured using the ultrasonic instrument, with each point being measured 10 times individually. The average value for each point was then calculated from the 10 measurements. The values annotated with yellow numerals in the figure corresponded to the average ultrasonic wave velocities of the various wall surfaces presented in Table 2, which signified the overall mean wave velocity levels across the five wall surfaces. The degree of ultrasonic testing points within each group reflected the variability of wave velocities at different locations on the same wall surface, greater height disparities among the colored column chart indicated more heterogeneous weathering conditions across different positions on that wall surface. Based on the analysis of Figure 9 in conjunction with the on-site deterioration features survey data, it was determined that the wave velocities of the measuring points on the east wall were generally elevated, with the majority of points substantially exceeding those of other zones, suggesting that the east wall possessed a relatively sound overall structure, with only sporadic and localized defects, and a low probability of large-scale deterioration features. Conversely, the majority of measuring points on the south wall exhibited consistently low wave velocities, indicating that this wall had undergone the most severe weathering, which was not confined to localized areas. Within each individual zone, the colored column chart displayed considerable height fluctuations, demonstrating that the distribution of deterioration features across a single wall surface was heterogeneous, with both well-preserved sectors and loosened sectors coexisting.

In accordance with calculations derived from the national standard “Code for investigation of geotechnical engineering” [25], the weathering grade of sandstone at the Yungang Grottoes is determined by the ratio of the longitudinal wave velocity of weathering rock to that of fresh rock. The correlation between the wave velocity ratio and the extent of weathering is presented in Table 3 [26,27]. All ultrasonic tests on Cave 29 sandstone were carried out under in situ ambient moisture conditions of the grotto site. The velocity-ratio-based evaluation framework largely mitigated systematic deviations originating from test configuration and rock-moisture variation, because both the numerator (weathered rock velocity) and denominator (fresh-rock reference velocity) were subject to comparable environmental-induced velocity shifts. This ratio-driven approach guaranteed the comparability of the classification thresholds with our in situ experimental conditions. The mean ultrasonic pulse wave velocity measured on the east wall of Cave 29 was 1928 m/s, which was significantly higher than that recorded on the other wall surfaces, and was classified as Grade 3 moderate weathering. As revealed by the distribution of colored column chart measurements in Figure 9, a substantial number of measuring points on the east wall exhibited wave velocities exceeding 1778 m/s, with some points attaining velocities above 2371 m/s, corresponding to the slight weathering grade. Only a minority of points registered values below 1778 m/s, falling within the strong weathering category. In general, the east wall was predominantly subject to moderate weathering, while certain local sectors demonstrated favorable preservation conditions (slight weathering), with only isolated scattered patches showing evidence of enhanced weathering. Among all the wall surfaces within the cave, the east wall possessed the optimum structural integrity and sustained the minimum degree of weathering deterioration feature. The mean ultrasonic pulse wave velocity recorded on the west wall was 1683 m/s, which fell below the threshold of 1778 m/s and was therefore classified as Grade 4 strong weathering. As revealed by the distribution of ultrasonic pulse wave velocity measuring points in Figure 9, the dispersion of data on the west wall was the greatest among all wall surfaces of the cave: the maximum measured value approached 2450 m/s (slight weathering), whereas the minimum dropped below 1185 m/s, nearing the lower bound of complete weathering. A comprehensive evaluation indicated that well-preserved rock masses and severely loosened rock masses were intermingled in an alternating distribution on the west wall.

The mean ultrasonic pulse wave velocity measured on the north wall was 1607 m/s, which was classified as Grade 4 strong weathering. This mean value fell within the strong weathering interval and was marginally lower than that recorded for the west wall. As revealed by the distribution of measuring points in Figure 9, the majority of points on the north wall were concentrated within the range of 1450 to 1850 m/s, with a limited number of points extending upward to the moderate weathering threshold, while no exceptionally low-value points were detected. The mean ultrasonic pulse wave velocity recorded on the ceiling was 1469 m/s, which was classified as Grade 4 strong weathering. This mean value was positioned within the middle portion of the strong weathering interval. The measuring points exhibited considerable scatter in their distribution, a phenomenon that was attributable to multiple factors, including prolonged exposure of the ceiling rock mass to self-weight stress, diurnal variations in temperature and humidity, condensation water infiltration, and the presence of extensive hollowing zones. The south wall yielded a mean ultrasonic pulse wave velocity of 1378 m/s, also falling within the Grade 4 strong weathering category. This value represented the lowest mean wave velocity among all wall surfaces of the cave and was situated near the lower bound of the strong weathering range. As was clearly evident from the colored column chart in Figure 9, virtually no measuring points on the south wall exceeded 1778 m/s, and no extensive sectors of moderate weathering with sound preservation were identified. A comprehensive evaluation indicated that the south wall had experienced prolonged and pervasive strong weathering, the south wall was determined to be the zone demanding the highest priority in terms of deterioration feature prevention and conservation management within Cave 29. Some weathering areas on the east wall, west wall, and ceiling had pulse wave velocities below 1000 m/s, reaching the Grade 5 complete weathering criterion.

The weathering grade of Cave 29 in the Yungang Grottoes was accurately measured using ultrasonic pulse velocity, providing scientific data for the subsequent targeted protection of cultural relics. The results (Table 2) indicate substantial variations in the measurements obtained from different positions on distinct walls, attributable to the internal structure of the measurement points. The average wave velocity in the ceiling, the lower and upper-middle sections of the north wall, and the upper niche on the east wall of Cave 29 is comparatively low, indicating that their internal structures are relatively loose, accompanied by deterioration features such as hollowing and exfoliation. The average wave velocity of the lower niche on the east wall is relatively larger, indicating that its internal structure is dense and free from hollowing deterioration feature. Overall, the ultrasonic wave velocities measured on the various wall surfaces of Cave 29 showed significant variations, with the spatial distribution of deterioration feature demonstrating marked heterogeneity. The rock mass integrity on the eastern side of the cave was found to be superior to that on the southern side and the ceiling, a phenomenon that was hypothesized to be closely associated with differences in cave orientation, duration of solar exposure, rainwater infiltration pathways, and ventilation conditions. In future investigations, the integration of environmental monitoring data could be undertaken to further clarify the correlative relationships between micro-environmental conditions and rock weathering, as well as wave velocity attenuation.

Ultrasonic pulse wave velocity served as an indicator of the internal integrity of the rock mass over a certain depth interval, whereas Leeb hardness characterized the surface mechanical properties of the rock. The results obtained from these two testing techniques did not fully correspond with each other, indicating that the weathering of the sandstone in Cave 29 displayed pronounced vertical heterogeneity. Ultrasonic pulse wave velocity served as an indicator of the internal integrity of the rock mass over a certain depth interval, whereas Leeb hardness characterized the surface mechanical properties of the rock. The results obtained from these two testing techniques did not fully correspond with each other, indicating that the weathering of the sandstone in Cave 29 displayed pronounced vertical heterogeneity. This discrepancy implied that weathering damage was more severe at the near-surface layer, while the inner rock matrix retained relatively better mechanical performance. Such vertical variation should be considered when evaluating the overall deterioration state of the grotto rock mass. In certain zones, the deterioration feature of the surface layer and that of the deeper portions were observed to be asynchronous, which adequately substantiated that the integrated utilization of multiple testing methods was indispensable for a comprehensive evaluation of the weathering condition of the grotto rock mass.

### 3.3. Cracks

**Figure 10:**
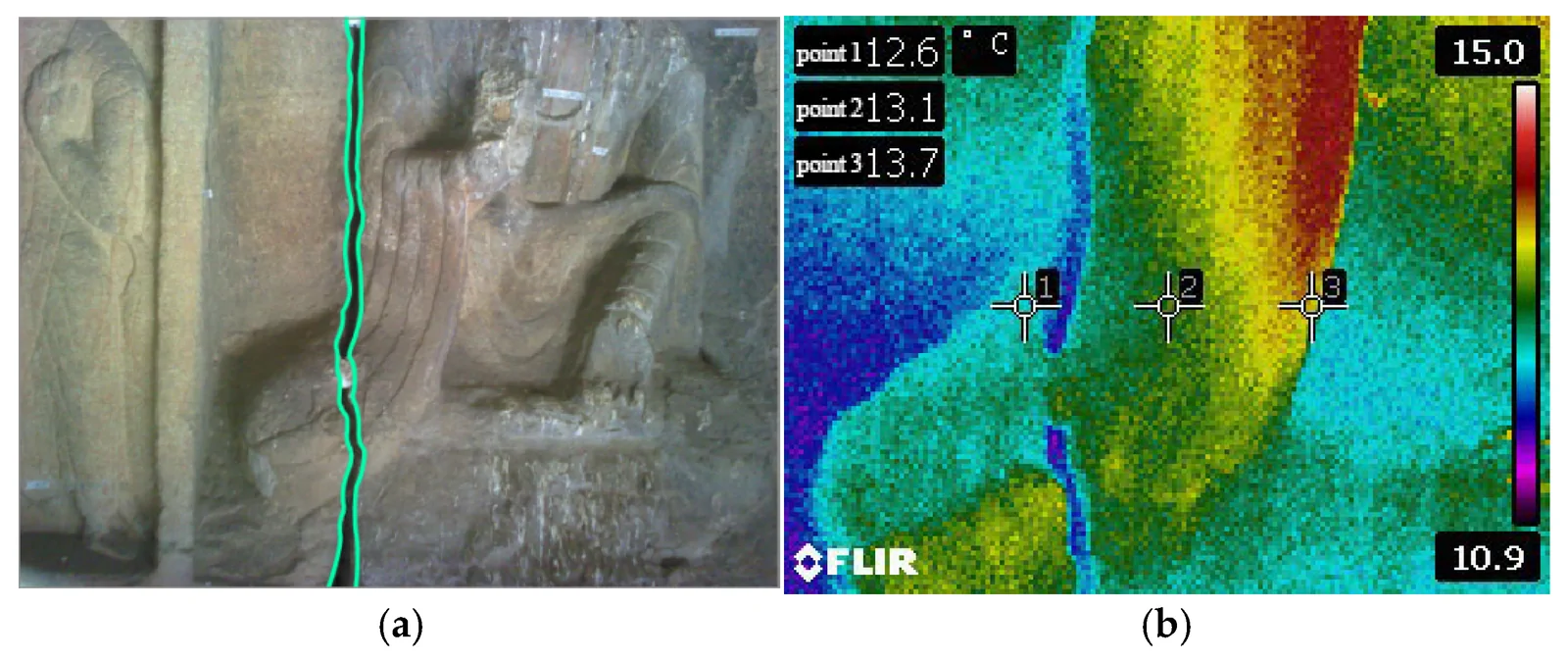
(**a**) Crack on the east wall of Cave 29. (**b**) Infrared thermal imager diagram on the east wall of Cave 29. Figure 10a was photographed by Miao Tian of Yungang Academy.

Through field investigation, it was found that the crack length of Cave 29 in the Yungang Grottoes was 15.81 m, among which the crack lengths on the ceiling, the south wall and the west wall were 4.07 m, 3.58 m, and 3.49 m, respectively. The ceiling exhibited a total of 28 cracks, constituting 51.85% of the total cracks within the cave. Specifically, the east wall had 12 cracks, the south wall had 6 cracks, the north wall had 5 cracks, and the west wall had 3 cracks. In the case of stone cultural relics exhibiting cracks, the air temperature within these cracks remained relatively low, manifesting as blue in infrared thermal imager, as depicted in Figure 10. The coloration gradually intensifies from the edges towards the center, thereby revealing the relative depth of the cracks. Figure 10a,b illustrated the fracture map and infrared thermal image of the east wall of Cave 29. Figure 10b showed that the infrared thermography clearly identified the fractures forming a belt-like zone, with temperatures lower than the adjacent intact areas. A crack was observed extending from the upper left edge of the Buddha niche to the left shoulder of the principal image within the Buddha niche. The crack had a trace length of 1.0 m, an aperture width of 5.0 mm, and was oriented in a near-vertical direction. The crack assessment was limited to surface trace geometry.

### 3.4. Surface Salt Efflorescence

**Figure 11:**
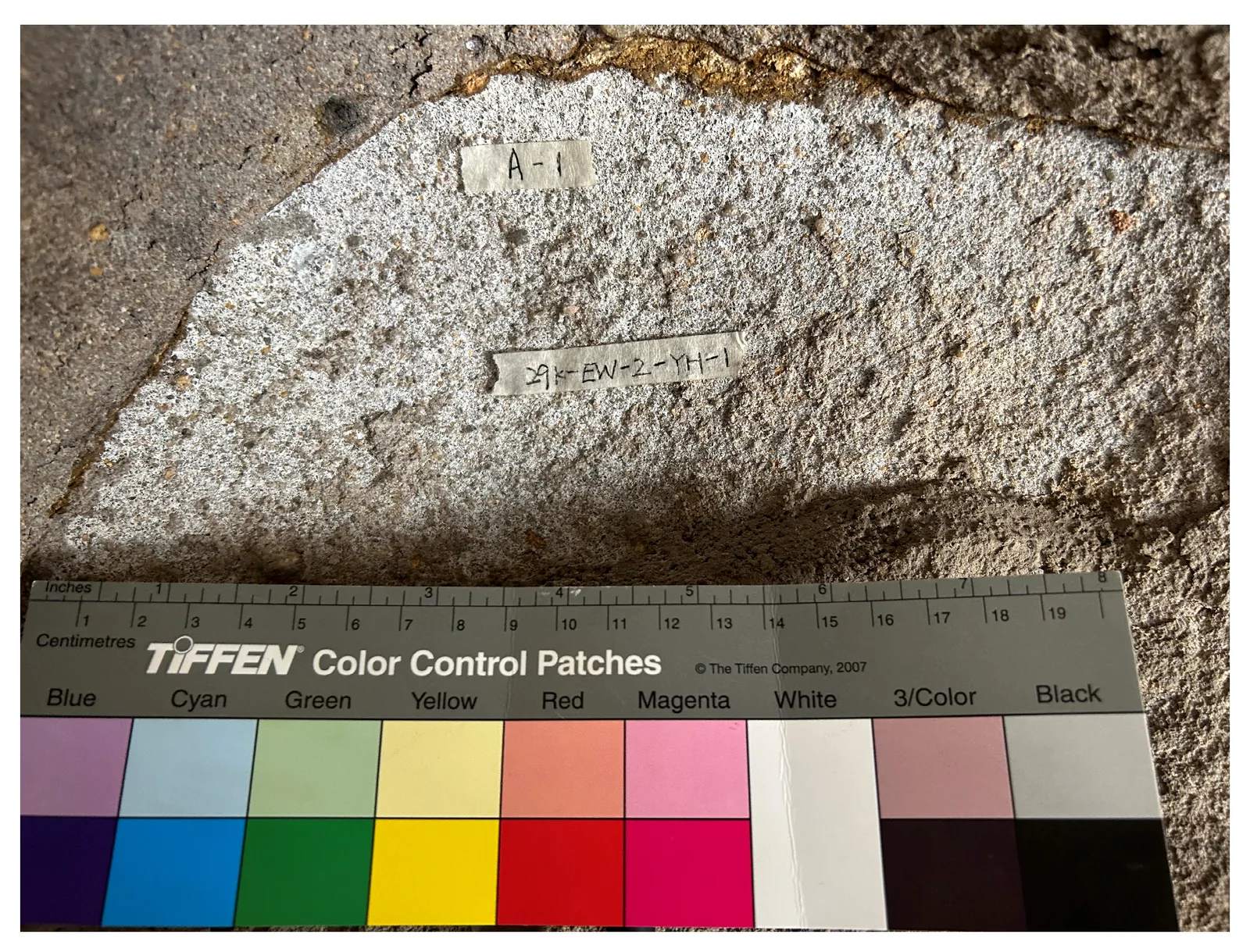
Surface salt efflorescence on the east wall of Cave 29. Figure 11 was photographed by Miao Tian of Yungang Academy.

**Figure 12:**
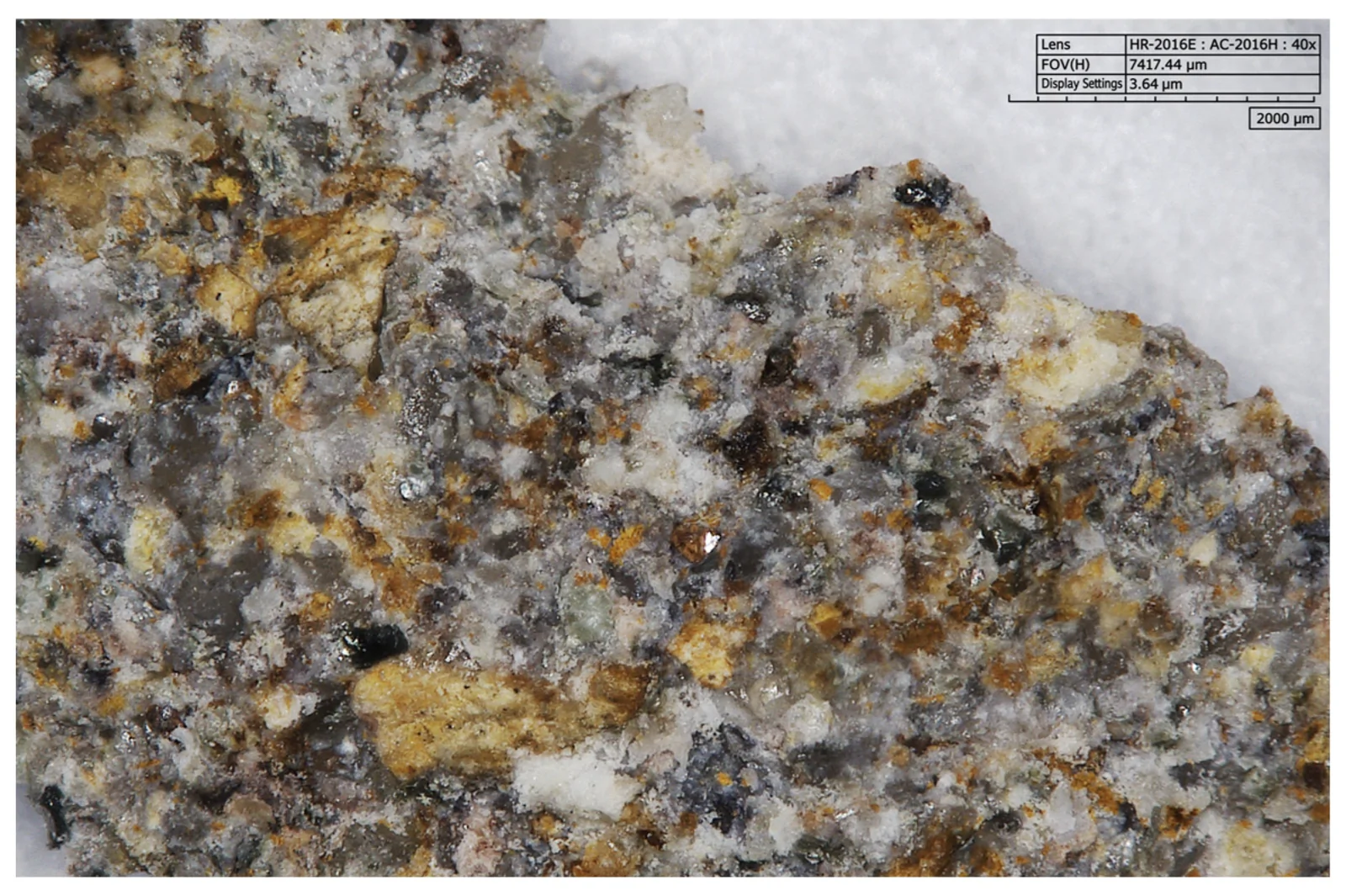
Surface salt efflorescence with ultra-depth field microscope.

Surface salt efflorescence denoted the phenomenon in which soluble salts accumulated and precipitated on the surfaces of stone cultural relics driven by capillary water movement. At that time, only one single surface salt efflorescence area (Figure 11) was identified at the northeast corner of the east wall of Cave 29, measuring 180 mm in width and 170 mm in height. From this observation, it could be inferred that the Yungang Academy had previously performed thorough cleaning of surface salt efflorescence in the Yungang Grottoes, which highlighted the necessity of regular maintenance. Observations using an ultra-depth field microscope revealed that a layer of white granular substances was enriched on the surface of the salt sample collected on site (Figure 12). Widespread white microcrystalline soluble salts crystals developed on sample surfaces. The salts were heterogeneously distributed and filled pores in agglomerated and flocculent forms. Salt crystals precipitated along mineral intergranular pores. Salt crystallization weakened interparticle cementation and loosened the rock skeleton. Salt crystals coexisted with yellowish-brown secondary iron oxide minerals, indicating that capillary water in pores transported salts and metal ions, followed by evaporation and precipitation.

The ion content associated with surface salt efflorescence at the northeast corner of the east wall in Cave 29 was determined using an ion chromatograph. The predominant component of surface salt efflorescence identified at this site was SO_4_^2-^, and the concentration of soluble salts ions was 4.114 mg·L^−1^. The enriched white granular substances observed under the ultra-depth-of-field microscope represented crystalline precipitates of soluble sulfate (SO_4_^2-^) quantified by ion chromatography (IC) on the stone surface, commonly referred to as surface salt efflorescence. The surface salt efflorescence occurred at the northeast corner of the east wall, specifically at the junction of the east wall, north wall, and ceiling, suggesting the potential presence of water leakage at this site. Guan et al. [28] proposed that the water seepage in Cave 2 of the Yungang Grottoes was replenished through atmospheric precipitation, and the measured SO_4_^2-^ content at the water seepage point was 136.3mg/L. The SO_4_^2-^ concentration at the northeast corner of the eastern wall in Cave 29 was 4.114 mg/L, constituting a mere 3% of the SO_4_^2-^ concentration observed in the seepage water of Cave 2.

The surface salt efflorescence at the northeast corner of the east wall in Cave 29 was analyzed using p-XRF. The analysis results were shown in Table 4. Based on the analytical results, the chlorine content was determined to be 1.57%. This soluble chloride tends to migrate with internal moisture toward the stone surface, where its crystallization exerted significant physical stress, serving as a primary contributor to sandstone powdering and exfoliation. The sulfur content was recorded at 7.92%. Given the absence of elemental oxygen in the detected form, this sulfur signal was attributed to sulfate species (SO_4_^2−^), which are widely recognized as one of the most hazardous soluble salts in stone cultural heritage. The cyclic deliquescence and recrystallization of sulfates within the pore network would generate substantial volumetric expansion, leading to progressive microstructural deterioration features. The calcium content was measured at 7.59% and exhibited a strong positive correlation with sulfur, implying that the predominant sulfate phase was likely calcium sulfate. Meanwhile, aluminum (3.1%) and silicon (8.55%), as diagnostic elements of aluminosilicate minerals (e.g., feldspars and clay components), remained relatively stable throughout the dataset. These elements served as internal references, suggesting that no extensive chemical replacement or substantial alteration of the original mineral matrix had occurred.

Considering the background interference from indigenous rock-forming minerals such as feldspar and calcite, the measured Ca/S molar ratio of approximately 1:1.30 (based on 7.59% Ca and 7.92% S) deviated from the stoichiometric value of 1:1 by about 30%. Given the semi-quantitative nature of portable XRF analysis on heterogeneous wall surfaces and the possible contribution of Ca from calcite or other Ca-bearing phases, this ratio provided suggestive but not conclusive evidence for the presence of calcium sulfate (CaSO_4_). Further analyses (e.g., XRD or Raman spectroscopy) would be beneficial for phase confirmation. This identification was corroborated by ion chromatography, which unambiguously detected SO_4_^2−^ as the dominant soluble anion, while elevated concentrations of NO_3_^−^ or F^−^ were not observed, thereby excluding the interference of other aggressive salt types such as nitrates. Given that the Yungang Grottoes sandstone was characterized by abundant carbonate cements (primarily CaCO_3_), the infiltration of acid rain or sulfuric acid (derived from atmospheric SO_2_ oxidation) would inevitably trigger neutralization reactions with the carbonate matrix. This pathway represented the most commonly documented mechanism for the formation of gypsum crusts on stone monument surfaces. Furthermore, microscopic examination using a digital microscope revealed white granular deposits exhibiting morphological features typical of gypsum crystals, consistent with previous descriptions [29,30]. Collectively, probable interpretation based on indirect evidence supported the conclusion that the soluble salts phase in the studied area could be tentatively assigned to calcium sulfate (CaSO_4_).

**Figure 13:**
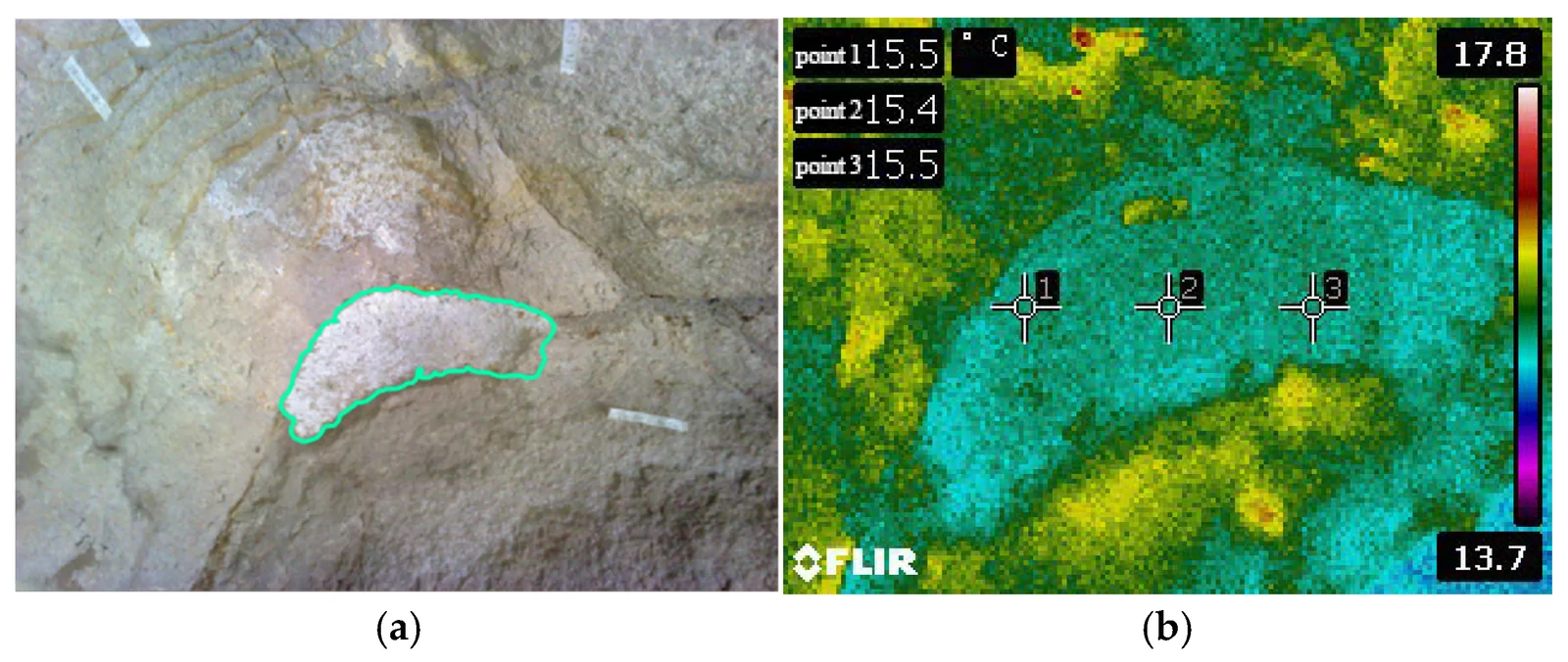
(**a**) Surface salt efflorescence at the east wall of Cave 29. (**b**) Infrared thermal imager diagram of surface salt efflorescence. Figure 13a was photographed by Miao Tian of Yungang Academy.

Infrared thermal imager was employed to analyze the spatial characteristics of surface salt efflorescence site. The results showed that the surface temperature of surface salt efflorescence area was significantly lower than that of the surrounding rock mass, suggesting either a high moisture content or an elevated salt concentration. The surface salt efflorescence location, as indicated in Figure 13a, was situated at the junction between the ceiling and the eastern wall, where a substantial presence of water rust was visually observed. This observation indicated that water seepage existed in this area. The combination of seepage water and soluble salts facilitated salt evaporation. This endothermic process absorbed heat from the surrounding environment, thereby resulting in a lower surface temperature at surface salt efflorescence wall. The IC analysis further revealed a relatively low concentration of SO_4_^2−^ at this location, which implied that the low-temperature anomaly captured by the infrared thermal imager might have originated from the migration and accumulation of salts, accompanied by capillary water transport. This process led to a localized moisture accumulation phenomenon, corresponding to a higher water content within the affected zone. By employing multi-technique testing techniques, including infrared thermography, the presence of hollowing (detachment) was identified in the surrounding walls. It was hypothesized that capillary water might have penetrated into the adjacent areas, promoting the continuous dissolution and reprecipitation of soluble salts. This cyclic physicochemical process progressively undermined the internal rock mass structure, ultimately resulting in the observed hollowing phenomenon.

The combination of microscopic observation, IC quantification, and X-ray fluorescence (XRF) elemental analysis appeared to provide a coherent line of evidence, which tended to support our understanding of the surface salt efflorescence at the northeastern corner of the east wall of Cave 29, while also pointed to its potential risks. Digital microscopy tended to offer direct morphological evidence, while IC and XRF appeared to furnish suggestive chemical and elemental fingerprints. The convergence of these independent lines of evidence indicated that this specific location was likely undergoing an active cycle of salt migration and crystallization. Based on the comprehensive assessment, the site was determined to be in an active phase of physical deterioration feature, presenting a high-risk deterioration feature condition that necessitates targeted conservation measures. Accordingly, a suite of preventive and remedial actions was proposed for areas affected by surface salt efflorescence. These include proactive desalination treatment, enhanced environmental monitoring, and in-depth subsurface investigation. Since surface salt efflorescence is intimately linked to concurrent weathering processes, it is recommended that desalination be performed in combination with consolidation treatments. Furthermore, a thorough inspection for potential water infiltration or leakage is advised, as the presence of seepage water is considered a critical factor perpetuating the ongoing cycle of salt efflorescence.

## 4. Conclusions

As a World Cultural Heritage site, the Yungang Grottoes suffered from various types of deterioration features under the impacts of natural environments and human activities. In this study, field investigation and multi-technique testing were adopted to conduct deterioration feature surveys and analyses on Cave 29 of the Yungang Grottoes, and the results were listed as follows:

(1) On the basis of deterioration feature investigations, this study carried out a comprehensive assessment of the distribution locations and deterioration features conditions of hollowing, weathering, cracks and surface salt efflorescence in Cave 29 of the Yungang Grottoes. Field surveys revealed that the total hollowing area inside Cave 29 reached 3.31 m^2^. Eighteen hollowing areas were identified on the cave ceiling, covering an area of 2.772 m^2^ and accounting for 83.75% of the total hollowing area of Cave 29. A severely weathering zone appeared on the head and upper torso of the Buddha niche in the lower layer of the northern cave wall (Figure 7). Groove-shaped weathering areas and hole-shaped weathering areas were observed on the south wall and east wall. Field investigations measured the total trace length of cracks in Cave 29 was measured at 15.81 m. 28 cracks existed on the cave ceiling, which made up 51.85% of all cracks within the cave. Only one surface salt efflorescence area was discovered at the northeastern corner of the eastern wall of Cave 29 (Figure 11), with a width of 180 mm and a height of 170 mm.
(2) To further analyze the formation causes and preservation conditions of the deterioration features, infrared thermal imagers were adopted to characterize hollowing, cracks and surface salt efflorescence, and the formation mechanisms of the three types of deterioration features were analyzed accordingly. The infrared thermal imager identified the distribution areas of hollowing, cracks and surface salt efflorescence. According to Leeb hardness tests, the average hardness values on all walls of Cave 29 ranged from 283 HLD to 308 HLD, all significantly lower than that of intact fresh rock (661.4 HLD), indicating weathering-induced surface hardening loss. The *data* analysis of Cave 29 walls revealed that the eastern wall had the highest average velocity (1928 m/s), indicating the best rock integrity, while the southern wall recorded the lowest (mostly below 1778 m/s), reflecting extensive weathering. Accordingly, the southern wall was identified as the top priority for deterioration control. Surface salt efflorescence site was discovered at the northeastern corner of the eastern wall in Cave 29. Microscopic observation, IC quantification and XRF elemental analysis tended to support our understanding of actual state and potential hazards of surface salt efflorescence at the northeastern corner of the eastern wall in Cave 29 of the Yungang Grottoes.

Field investigation and multi-technique analytical techniques were adopted to ana- lyze the preservation conditions and formation mechanisms of the four types of deterioration features in Cave 29. This study explored effective quantitative characterization methods for rock deterioration features based on multi-technique testing technologies, and provided scientific data support for the future conservation of stone cultural relics.

## Figures and Tables

**Table 1:** The test results of the Leeb hardness in Cave 29 of the Yungang Grottoes.

Wall	*n*	Mean ± SD (m/s) (HLD)	Range (Min–Max)
East wall	40	283 ± 38	164–367
West wall	24	293 ± 71	163–407
South wall	33	295 ± 30	257–368
North wall	63	283 ± 41	182–396
Ceiling	18	308 ± 19	268–344

Table note: HLD = Leeb hardness value in HLD scale; *n* = number of effective measurement points; SD = standard deviation.

**Table 2:** Ultrasonic testing results on different walls of Cave 29.

Wall	*n*	Mean ± SD (m/s)	Range (Min–Max)
East wall	10	1928 ± 478	875–2464
West wall	10	1683 ± 415	1101–2456
South wall	10	1379 ± 81	1245–1475
North wall	10	1608 ± 195	1244–1874
Ceiling	9	1469± 329	989–2008

**Table 3:** Classification criteria for wave velocity and weathering grade of weathering sandstone in the Yungang Grottoes.

National Standard			Weathering Degree	Describe
Speed (m/s)	Speed Ratio	Grade		
0~593	<0.2	6	Residual soil	Recently deposition, including residual soil.
593~1185	0.2~0.4	5	Complete Weathering	The structure was almost completely destroyed, accompanied by highly developed weathering and cracks.
1185~1778	0.4~0.6	4	Strong Weathering	The structure was predominantly damaged, accompanied by well-developed weathering and cracks.
1778~2371	0.6~0.8	3	Moderate Weathering	The structure was partially damaged, accompanied by the development of weathering and cracks.
2371~2667	0.8~0.9	2	Slight Weathering	The structure remained basically unchanged, accompanied by minor weathering and cracks.
2667~2963	0.9~1	1	Unweathering	The rock core was fresh, accompanied by occasional signs of weathering and cracks.

**Table 4:** Results of p-XRF analysis on surface salt efflorescence to the east wall of Cave 29 (%).

Location	Cl	Al	Si	S	Ca
The northeast corner of the east wall	1.57	3.1	8.55	7.92	7.59

## Data Availability

The original contributions presented in this study are included in the article. Further inquiries can be directed to the corresponding authors.

## References

[1] Su B. (1996). The Cave Temples of China.

[2] Chen Q. (2007). Shui Jing Zhu Collated and Annotated.

[3] Fu Z., Peng T., Li Y., Wu W., Zhang G. (2006). Analysis of water seepage mechanism in the weathering conservation of the Yungang Grottoes stone carvings. Proceedings of the 2005 Yungang International Academic Symposium Conservation Volume.

[4] Qu Y., Huang K., Xu X., Wu Z. (1988). A Study on the powdery deposits on the surface and subsurface layers of stone carvings in the Yungang Grottoes of Datong and their role in the weathering of the carvings. Selected Proceedings of the Third National Engineering Geology Conference.

[5] Sun Y., Yan H., Zhang Z. (2018). The deterioration feature investigation and protection study of Lubanyao Grotto. J. Natl. Mus. China.

[6] Sun M., Cao Z., Shen Y., Shang X., Luo Y. (2024). Study on the regional characteristics of sandstone grotto deterioration in the Longdong area. Sci. Conserv. Archaeol..

[7] Sun Y., Fu Z. (2020). Research on deterioration feature prevention and protection techniques of northern Grottoes: A case study of Jiaoshan Temple Grottoes in Datong. North. Cult. Relics..

[8] Huang J., Wang J., Gao F., Wang F., Qi Y., Liu S. (2018). Recent progresses in sandstone cave temples conservation: A case study of Yungang Grottoes. Southeast Cult..

[9] Li H., Sun Z., Shi Y., Yang C., Li B. (2006). Analysis of dust deposition on the stone carvings surface in the Yungang Grottoes. Proceedings of the 2005 Yungang International Academic Symposium Conservation Volume.

[10] Wang J., Xu J., Gong M., Wang Y. (2021). Characterization of weathering in the sandstone of the Yungang Grottoes based on SEM images and microscopic seepage model. Hydrogeol. Eng. Geol..

[11] Zhang L. (2023). Application analysis of ultrasonic non-destructive testing technology in the protection of Yungang Grottoes. Ident. Apprec. Cult. Relics.

[12] Liu R., Zhang B., Zhang H., Shi M. (2011). Deterioration of Yungang Grottoes: Diagnosis and research. J. Cult. Herit..

[13] Zhang S. (2019). Characteristics of Air Pollution in Yungang Grottoes and Its Role in Sandstone Weathering. Master’s Thesis.

[14] Rihosek J., Bruthans J., Masin D., Filippi M., Carling G., Schweigstillova J. (2016). Gravity-Induced stress as a factor reducing decay of sandstone monuments in Petra, Jordan. J. Cult. Herit..

[15] Wedekind W., Gross C., Hoffmann A., Siegesmund S. (2018). Damage phenomena and petrophysical properties of sandstones at the Phnom Bakheng Temple (Angkor, Cambodia): First investigations and possible conservation measures. Environ. Earth Sci..

[16] Gomez-Heras M., Benavente D., Pla C., Martinez-Martinez J., Fort R., Brotons V. (2020). Ultrasonic pulse velocity as a way of improving uniaxial compressive strength estimations from Leeb hardness measurements. Constr. Build. Mater..

[17] Fort R., Buergo M., Perez-Monserrat E., Varas M. (2010). Characterisation of monzogranitic batholiths as a supply source for heritage construction in the northwest of Madrid. Eng. Geol..

[18] Liu C., Zhang S., Ding L., Liao J. (2012). Identification of dangerous rock mass of high slope and study of anchoring method based on laser scanning. Chin. J. Rock Mech. Eng..

[19] Hong J., Zhang Y., Chen H., Zhang S., Cheng Y., Yan H., Du Y., Wang J., Huang J. (2026). Quantitative assessment of sandstone weathering based on multi-data combination of nondestructive testing in the Yungang Grottoes, China. Int. J. Archit. Herit..

[20] Mammoliti E., Cupido M., Teloni R., Tittarelli F., Giuli G., Paris E., Farabollini P., Santini S. (2024). Implementation of a Non-Destructive Method to Assess Weathering Deterioration of Sandstones in Cultural Heritage. Bull. Eng. Geol. Environ..

[21] Zhang Z. (2017). The Complete Works of Yungang Grottoes.

[22] Peng M. (2017). The Construction Project of the Yungang Grottoes.

[23] (2008). Classification and Legend on the Deterioration of Stone Cultural Relics.

[24] Hou Z., Zhe R., Zhang Z. (2018). Estimating the weathering degree of rock surface of cultural relic from Leeb hardness for nondestructive measurement. J. Eng. Geol..

[25] (2009). Code for Investigation of Geotechnical Engineering.

[26] Meng T. (2014). Comprehensive Research Analysis on Weathering of Yungang Grottoes. Doctoral Dissertation.

[27] Ding H. (2017). Stability Analysis of Sandstone Columniation Based on Ultrasonic Detection. Master’s Thesis.

[28] Guan C., Yan H., Feng W., Wang S., Lu J., Fan X., Wang H., Feng F. (2020). Determination of anions in water samples from Yungang Grottoes area by ion chromatography. J. Shanxi Datong Univ. (Nat. Sci. Ed.).

[29] Jafarzadeh A., Burnham C. (1992). Gypsum crystals in soils. J. Soil Sci..

[30] Watson A. (1988). Desert gypsum crusts as palaeoenvironmental indicators: A micropetrographic study of crusts from southern Tunisia and the central Namib Desert. J. Arid Environ..

